# Error propagation analysis of seven partial volume correction algorithms for [^18^F]THK-5351 brain PET imaging

**DOI:** 10.1186/s40658-020-00324-9

**Published:** 2020-09-14

**Authors:** Senri Oyama, Ayumu Hosoi, Masanobu Ibaraki, Colm J. McGinnity, Keisuke Matsubara, Shoichi Watanuki, Hiroshi Watabe, Manabu Tashiro, Miho Shidahara

**Affiliations:** 1 Division of Cyclotron Nuclear Medicine, Cyclotron and Radioisotope Center, Sendai, Japan; 2 Division of Applied Quantum Medical Engineering, Department of Quantum Science and Energy Engineering, Graduate School of Engineering, Tohoku University, Sendai, Japan; 3 Department of Radiology and Nuclear Medicine, Research Institute for Brain and Blood Vessels, Akita Cerebrospinal and Cardiovascular Center, Akita, Japan; 4 School of Biomedical Engineering and Imaging Sciences, King’s College London, London, UK; 5 King’s College London and Guy’s and St Thomas’ PET Centre, St Thomas Hospital, London, UK; 6 Division of Radiation Protection and Safety Control, Cyclotron and Radioisotope Center, Tohoku University, Sendai, Japan

**Keywords:** Partial volume correction, Brain PET, Error-propagation analysis, [^18^F]THK-5351

## Abstract

**Background:**

Novel partial volume correction (PVC) algorithms have been validated by assuming ideal conditions of image processing; however, in real clinical PET studies, the input datasets include error sources which cause error propagation to the corrected outcome.

**Methods:**

We aimed to evaluate error propagations of seven PVCs algorithms for brain PET imaging with [^18^F]THK-5351 and to discuss the reliability of those algorithms for clinical applications. In order to mimic brain PET imaging of [^18^F]THK-5351, pseudo-observed SUVR images for one healthy adult and one adult with Alzheimer’s disease were simulated from individual PET and MR images. The partial volume effect of pseudo-observed PET images were corrected by using Müller-Gärtner (MG), the geometric transfer matrix (GTM), Labbé (LABBE), regional voxel-based (RBV), iterative Yang (IY), structural functional synergy for resolution recovery (SFS-RR), and modified SFS-RR algorithms with incorporation of error sources in the datasets for PVC processing. Assumed error sources were mismatched FWHM, inaccurate image-registration, and incorrectly segmented anatomical volume. The degree of error propagations in ROI values was evaluated by percent differences (%diff) of PV-corrected SUVR against true SUVR.

**Results:**

Uncorrected SUVRs were underestimated against true SUVRs (− 15.7 and − 53.7% in hippocampus for HC and AD conditions), and application of each PVC algorithm reduced the %diff. Larger FWHM mismatch led to larger %diff of PVC-SUVRs against true SUVRs for all algorithms. Inaccurate image registration showed systematic propagation for most algorithms except for SFS-RR and modified SFS-RR. Incorrect segmentation of the anatomical volume only resulted in error propagations in limited local regions.

**Conclusions:**

We demonstrated error propagation by numerical simulation of THK-PET imaging.

Error propagations of 7 PVC algorithms for brain PET imaging with [^18^F]THK-5351 were significant. Robust algorithms for clinical applications must be carefully selected according to the study design of clinical PET data.

## Background

### Error propagations in PVC of PET images

Positron emission computed tomography (PET) enables quantification of the biodistribution of administrated radiopharmaceuticals in vivo. However, the PET images suffer from the partial volume effect (PVE) due to the limited spatial resolution of PET scanners, where regional depiction of the uptake of PET radiopharmaceutical is blurred and its quantitative accuracy is reduced. Correction of PVE, known as partial volume correction (PVC), is usually performed for PET images by using spread function of blurring and an anatomical prior during post-reconstruction processing or during image reconstruction [1].

Post-reconstruction PVC, together with anatomical information based on magnetic resonance (MR) image, has been distributed as commercial or academic software and used for clinical studies due to easy realization and recent advances of hybrid PET-MRI scanners [2, 3]. In general, the accuracy of post-reconstruction PVC algorithms has been validated under the ideal conditions of correctly assigned point spread function, accurately delineated region contours, and excellent registration of anatomical images with PET images. However, in real situations of clinical study, discrepancies from the ideal conditions can easily happen for example due to shift-variant spatial resolution on PET images [4], incorrect segmentation of regions due to parameters used for image processing or distortion of MR images itself, and inaccurate image registration between PET and MR images. Even a small amount of discrepancy is likely to influence the output PVE-corrected image, as a result of error propagation [5].

### The necessity of PVC for pathological AD-PET study

For the diagnosis of early Alzheimer’s disease (AD), the in vivo PET imaging of characteristic pathological features aggregated amyloid protein (Aβ) and neurofibrillary tangle (tau) by using dedicated radiopharmaceuticals has been recognized as potentially having an important role [6]. However, PVC in brain PET imaging for diagnosis of AD is still challenging [7]. Elderly subjects are expected to have regional brain atrophy with/without physiological change. Radiopharmaceutical uptake in atrophied cortical regions of elderly subject is more difficult to quantify due to more severe PVE than normal cortical regions in young subjects. Furthermore, some radiopharmaceuticals for Aβ and tau PET imaging have high non-specific binding in white matter and these spill into targeted grey matter regions leading overestimation of accumulations [8, 9]. The non-specific binding in white matter is not significant in studies of cerebral blood flow, glucose metabolism, and neuro-receptor imaging but in case of pathological Aβ and tau imaging is sometimes significant [9]. Therefore, in order to detect or diagnosis AD in the early phase, PVC is considered necessary [10].

There have been several reports which stated that application of PVC for of tau and amyloid PET imaging improved the accuracy and precision of the quantification of radiopharmaceutical-uptake [8–18]. Lopez-Gonzalez et al. investigated the effect of white matter spill-in on SUVRs by using the regional voxel-based (RBV) [7] and iterative Yang (IY) [1] PVC algorithms [9]. However, in many cases, the applied PVC methods were limited to the classical and popular Müller-Gärtner (MG) [19] and geometric transfer matrix (GTM) methods [20, 21] and similar algorithms. Recently, cross-sectional or longitudinal, brain network big data analysis of a PET database combined with PVC pipeline processing has been introduced into the PET field [18, 22]; however, the automatic processing pipeline has a potential to include and propagate errors accidentally resulting in incorrect conclusions. It is obvious that careful processing can prevent error propagations. Furthermore, the robustness of PVC algorithms against error propagation would be important to evaluate.

### Error propagation analysis of PVC algorithms

There have been several reports about error propagation of PVC for brain PET imaging. It was reported that the incorrect specification of the full-width of half-maximum (FWHM) parameter with wider or narrower spread function of blurring relative to the true spread function resulted in the systematic over or underestimation in the correction of simulated [^18^F]FDG images by eight PVC algorithms [3]. For simulated [^18^F]FDOPA images, PV correction by GTM algorithm together with inaccurate image registration between PET and MRI reduced the quantitative accuracy of radioactivity concentration in the region of interests (ROI) [21]. Furthermore, if individual anatomical images with inaccurate co-registration or inaccurately segmented anatomical regions are used for creation of an ROI template, these will have an effect on the ROI values after error-propagated PV correction. Due to the importance of PVC in pathological AD-PET study, there is a need of systematic error propagation analysis. In this study, we aimed to perform error propagation analysis of seven PVC algorithms using digital phantom simulations for pathological brain PET [^18^F]THK-5351 imaging.

Seven partial volume correction algorithms were MG, GMT, Labbé (LABBE) [23], RBV, and IY, structural functional synergy for resolution recovery (SFS-RR) [24], and modified SFS-RR, respectively. MG, GTM, RBV, and IY are popular algorithms implemented in commercial or academic software. LABBE was an algorithm which showed different recovery of PVE from GTM, RBV, and IY in clinical PET images of [^18^F]THK-5351 in our previous study [25]. SFS-RR and modSFSRR are quite different algorithms from the five former algorithms which we expected different error propagation property.

## Methods

### Digital phantom simulation

**Fig. 1:**
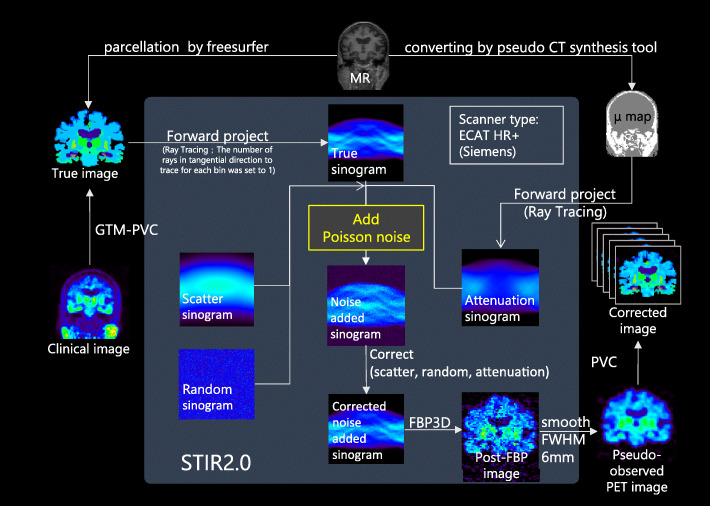
Schematic flow of the data processing

**Fig. 2:**
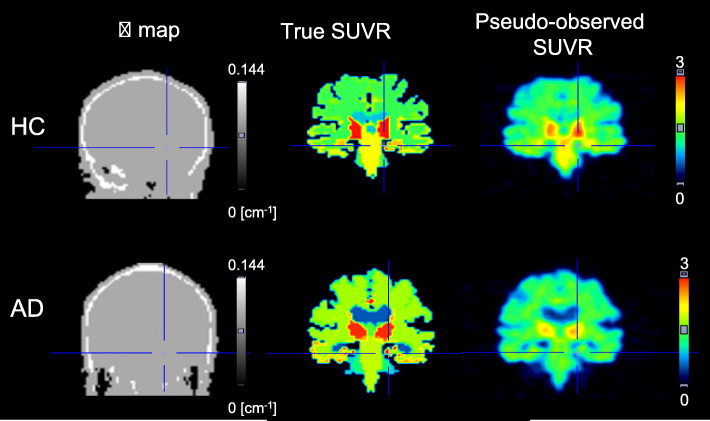
Simulated images of attenuation-map, true SUVR and pseudo-observed SUVR images

To validate error propagations during PVC processing, pseudo-observed [^18^F]THK-5351 SUVR images of each one healthy, elderly individual and one elderly individual with typical AD were numerically generated (Figs. 1 and 2). Detailed information regarding the original clinical data sets (separately acquired PET and MR images) was provided in our previous report [25]. After automated parcellation of individual T1-weighted MR image was implemented by the FreeSurfer neuroimage analysis software package with version 5.1 [26–29], the parcellation map was subdivided from original regions into 50 regions [25]. The individual observed PET images were corrected PVE by the GTM algorithm and then for individual regions of the parcellation map, the PV-corrected SUVR values were used for true SUVR images for each condition. The true SUVR images were not processed with any filtering. Pseudo-observed SUVR images for each condition were analytically simulated by using STIR 2.0 package [30] while assuming ECAT HR+ PET scanner. Forward-projected true SUVR images together with attenuation were combined with scaled Poisson noise, estimated random and scatter events, and then corrected for these random, scatter and attenuation. Finally, corrected sinograms were reconstructed with 3-dimensional filtered back projection with ramp filter and smoothed by 3-dimensional Gaussian filter with 6 mm FWHM to produce clinically realistic images [31]. Matrix size and pixel size were 128 × 128 × 63 (2.682 × 2.682 × 2.425 mm^3^ voxels). In our processing, scatter coincidences (estimated by single scatter simulation) and random coincidences (achieved by constant random background) were once added into the forward projected sinogram and subtracted from the noisy sinogram. The noise level of the image reconstructed from the noisy sinogram was finally adjusted with scaled Poisson noise to achieve a similar noise level to that of clinical PET images. The attenuation map of 511 keV gamma ray (*μ* = 0.096 cm^−1^ and 0.144 cm^−1^ for soft tissue and bone, respectively) was created by segmenting T1-weighted MR images with a pseudo-CT synthesis tool (http://niftyweb.cs.ucl.ac.uk/), and then the segmented images were carefully checked and manually re-drawn by an experienced physician (Fig. 2).

This study was approved by the Ethics Committee on Clinical Investigations of Tohoku University School of Medicine (No.2019-1-953) and was performed in accordance with the Declaration of Helsinki. Written informed consent was obtained from all subjects after a complete description of the study had been provided.

### Partial volume correction methods

Seven partial volume correction algorithms were implemented for pseudo-observed PET SUVR images. The detail of each algorithm is described in [24, 25] and here we try to give brief explanations. MG is a voxel-based method for grey matter regions with the assumption that uptake in white matter can be accurately represented by its mean value [19]. GTM is a ROI-based method by taking inverse of geometric transfer matrix, which represents the contribution of spillover from source region into target region [20, 32]. The LABBE [23] is also a ROI-based method by taking inverse of the matrix, which represents the contribution of spillover from source pixel into target region. The RBV [7] is an extension of the GTM and the voxel-wise correction of Yang et al. [33]. The iterative Yang (IY) [1] is a further adaptation of the Yang method [33], an iterative loop where an estimate of regional mean values is updated, based on current values. SFS-RR is image-based algorithm, which weights the functional versus structural information in the wavelet space [24]. Structural information was originally implemented as synthetic image assigned ROI values of PET image in each anatomical region. In this study, modified SFS-RR (mod SFS-RR) was also introduced by using PVC-GTM values rather than the ROI values of the PET image in the structural image.

### Data processing

PVC processing of MG, GTM, LABBE, RBV, and IY correction were implemented using the PETPVC toolbox (https://github.com/UCL/PETPVC) [28]. Ten iterations were performed for IY. SFS-RR and mod SFS-RR were performed by in-house software [24].

As error sources of PVC processing, mismatched FWHM, errors of image registration, and errors of anatomical volume segmentation were intentionally included. The FWHM of pseudo-observed images were estimated as 7 mm by using point object and fitting the Gaussian function, and therefore, 7 mm was set as the true FWHM. For PVC processing, 5, 6, 8, and 9 mm FWHMs (mismatch: − 2, − 1, + 1, + 2 mm) were used. Registration errors between PET and T1-weighted MR images, which can easily happen in clinical data, were implemented as − 5.4, − 2.7, + 2.7, and + 5.4 mm *X*-axis translations and − 4.9, − 2.4, + 2.4, and + 4.9 mm axial (Z) translations, respectively. We had two scenarios related to the anatomical segmentation error of MR images, (i) inaccurate delineation of hippocampus volume (by factors of 0.5, 0.7, 1.4, and 1.6) only and (ii) incorrect segmentation of cortical volume (by factors of 0.6, 0.8, 1.3, and 1.5). Realization of different volumes was done by shrinking or dilation of the regional anatomical labels while adjusting manually.

### Data analysis

PVC-SUVR images with [^18^F]THK-5351 were normalized with the ROI value of the cerebellar grey matter. For error propagation analysis, 6 ROIs (middle and inferior temporal cortex, parietal cortex, occipital cortex, parahippocampal gyrus, hippocampus, fusiform) were selected [34] and left and right for each regions were united, except for the cases of registration error with *X*-direction. The error propagation of the different seven PVC algorithms was evaluated by %difference of ROI value of PVC-SUVR image against that of true SUVR image as follows,

1 $$ \%\mathrm{difference}=\frac{{\mathrm{ROI}}_{\mathrm{PVC}}-{\mathrm{ROI}}_{\mathrm{true}}}{{\mathrm{ROI}}_{\mathrm{true}}}\times 100 $$

Note that in case of registration and segmentation errors, two types of ROI template with error and without error were used for ROI analysis.

## Results

### The impact of PVCs for ideal conditions

**Fig. 3:**
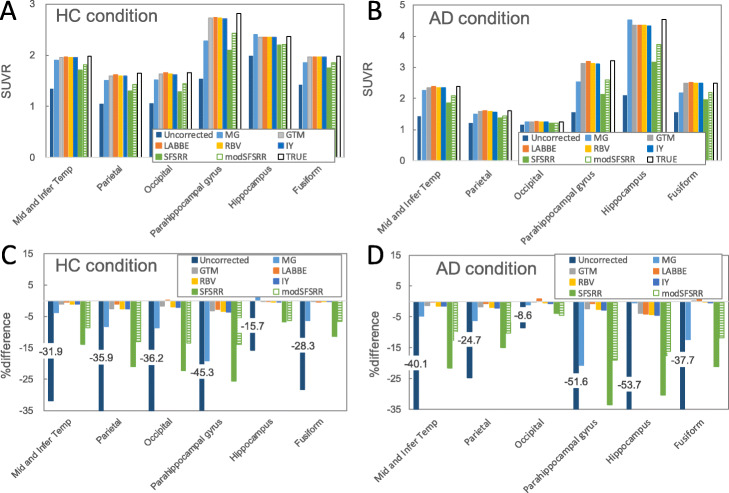
SUVRs and %difference of pseudo-observed/PVC-SUVRs against true SUVRs. **a** SUVRs in HC condition, **b** SUVRs in AD condition, **c** %difference in HC conditions, and **d** %difference in AD condition

As shown in Fig. 3, under ideal condition without any errors, uncorrected and PVC- SUVRs for 6 regions and 2 conditions (HC and AD) were compared against the true SUVR. For all regions and conditions, uncorrected SUVRs were underestimated against true SUVRs, and for all PVC algorithms, quantifications of SUVRs were improved.

### The error propagation of FWHM mismatch

**Fig. 4:**
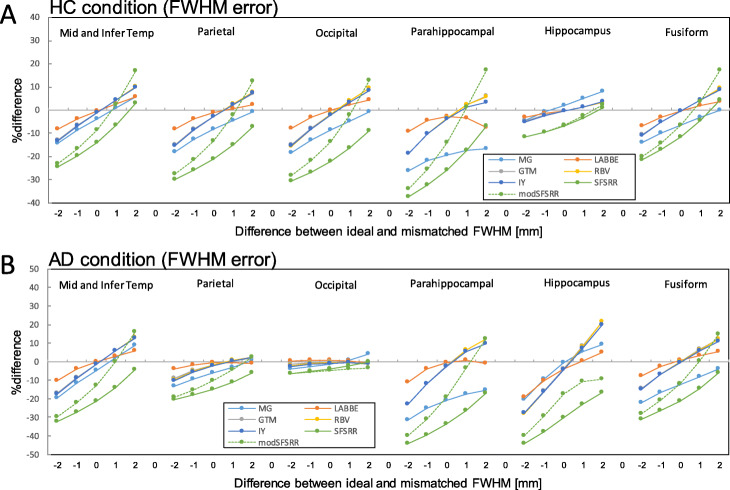
%difference of PVC-SUVRs with FWHM mismatch against true SUVRs. **a** HC condition and **b** AD condition

Figure 4 indicates %difference of PVC-SUVRs with FWHM mismatch against true SUVRs for HC and AD conditions. For an overall tendency, a larger FWHM mismatch led to larger %differences of PVC-SUVRs against true SUVRs. In the HC condition (Fig. 4a) with 1 mm difference of FWHMs, the maximum %differences observed were − 21.8 (para-hipp), − 4.5 (para-hipp), − 10.2 (para-hipp), − 10.2 (para-hipp), − 10.3 (para-hipp), − 32.2 (para-hipp), and − 25.4 (para-hipp) for MG, LABBE, GTM, IY, RBV, SFS-RR, and mod SFS-RR, respectively. In the AD condition (Fig. 4b) with 1 mm difference of FWHMs, the maximum %differences observed were − 25.3 (para-hipp), − 10.2 (hipp), − 15.7 (hipp), − 16.0 (hipp), − 16.1 (hipp), − 39.4 (para-hipp), and − 31.0(para-hipp) for MG, LABBE, GTM, IY, RBV, SFS-RR, and mod SFS-RR, respectively.

### The error propagation of imperfect registration

**Fig. 5:**
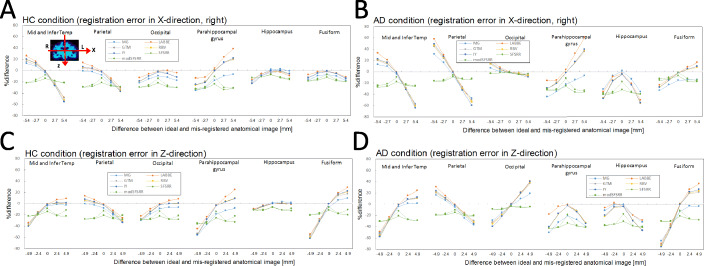
%difference of PVC-SUVRs with registration error (*X*) against true SUVRs. **a** HC condition and **b** AD condition and PVC-SUVRs with registration error (*Z*) against true SUVRs: **c** HC condition and **d** AD condition. ROI templates were from accurately registered anatomical images

Figure 5 indicates %difference of PVC-SUVRs with registration error of *X*- and *Z*-direction against true SUVRs for HC and AD conditions, where ROI templates were from accurately registered anatomical images. For an overall tendency, a larger difference between ideal and registered anatomical images led to larger %differences between PVC-SUVRs with registration error and true SUVRs. In the HC condition (Fig. 5a) with 2.7 mm (1 pixel) difference in *X*-direction, the maximum %differences observed were − 30.2 (para-hipp), 23.5 (para-hipp), − 23.1 (mid and infer temp), − 26.6 (mid and infer temp), − 25.6 (mid and infer temp), − 34.1 (para-hipp), and − 29.9 (para-hipp) for MG, LABBE, GTM, IY, RBV, SFS-RR, and mod SFS-RR, respectively. In the AD condition (Fig. 5b) with 2.7 mm (1 pixel) difference of registration in *X*-direction, the maximum %differences observed were − 35.1 (para-hipp), 32.4 (para-hipp), − 28.7 (mid and infer temp), − 33.1 (parietal), − 30.7 (mid and infer temp), − 38.2 (hipp), and − 35.7 (hipp) for MG, LABBE, GTM, IY, RBV, SFS-RR, and mod SFS-RR, respectively. In the HC condition with 2.4 mm (1 pixel) difference of registration in *Z*-direction (Fig. 5c), the maximum %differences observed were − 36.9 (para-hipp), − 27.4 (fusiform), − 28.6 (fusiform), − 31.6 (fusiform), − 30.6 (fusiform), − 35.7 (para-hipp), and − 32.9 (para-hipp) for MG, LABBE, GTM, IY, RBV, SFS-RR, and mod SFS-RR, respectively. In the AD condition with 2.4 mm (1 pixel) difference of registration in *Z*-direction (Fig. 5d), the maximum %differences observed were − 39.5 (fusiform), − 35.5 (fusiform), − 38.7 (fusiform), − 42.4 (fusiform), − 40.2 (fusiform), − 42.2 (para-hipp), and − 41.4 (para-hipp) for MG, LABBE, GTM, IY, RBV, SFS-RR, and mod SFS-RR, respectively.

**Fig. 6:**
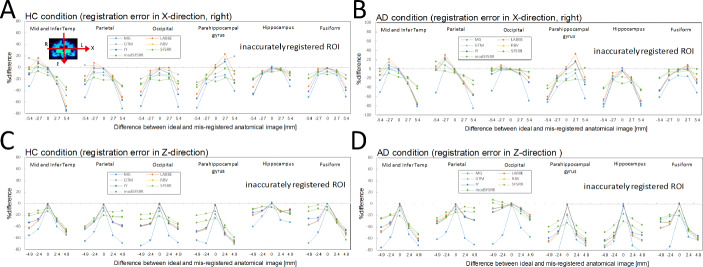
%difference of PVC-SUVRs with registration error (*X*) against true SUVRs. **a** HC condition and **b** AD condition and %difference of PVC-SUVRs with registration error (*Z*) against true SUVRs: **c** HC condition and **d** AD condition. ROI template was from inaccurately registered anatomical images

Figure 6 indicates %difference of PVC-SUVRs with registration error of *X*- and *Z*-direction against true SUVRs for HC and AD conditions, where ROI templates were from inaccurately registered anatomical images. Different tendencies between Figs. 5 and 6 were observed for seven PVC algorithms.

### The error propagation of incorrect segmentations

**Fig. 7:**
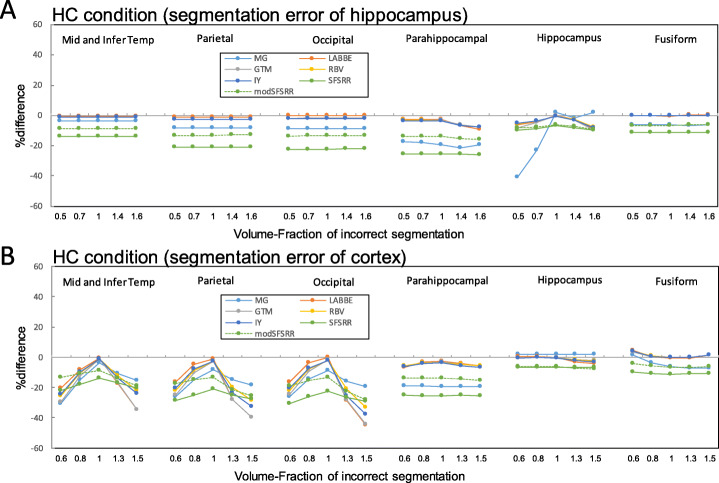
%difference of PVC-SUVRs with segmentation error against true SUVRs. **a** Local (hippocampus) and **b** global segmentation errors. ROI template was from correctly segmented anatomical images

Figure 7 indicates %difference of PVC-SUVRs with segmentation errors of hippocampus and of the global cortex against true SUVRs for the HC condition, where ROI templates were from correctly segmented anatomical images. Error propagations were only observed in limited local regions (hippocampus in Fig. 7a and peripheral cortex in Fig. 7b). In the case of 1.4 times larger hippocampus volume (Fig. 7a), the %differences observed in the hippocampus region were − 1.66, − 2.84, − 3.10, − 3.12, − 2.77, − 7.96, and − 7.32 for MG, LABBE, GTM, IY, RBV, SFS-RR, and mod SFS-RR, respectively. In the case of 0.8 times smaller cortical volume (Fig. 7b), the %differences observed in the mid and inferior temporal cortex were − 14.9, − 8.31, − 12.5, − 9.86, − 10.4, − 17.9, and − 10.7 for MG, LABBE, GTM, IY, RBV, SFS-RR, and mod SFS-RR, respectively.

**Fig. 8:**
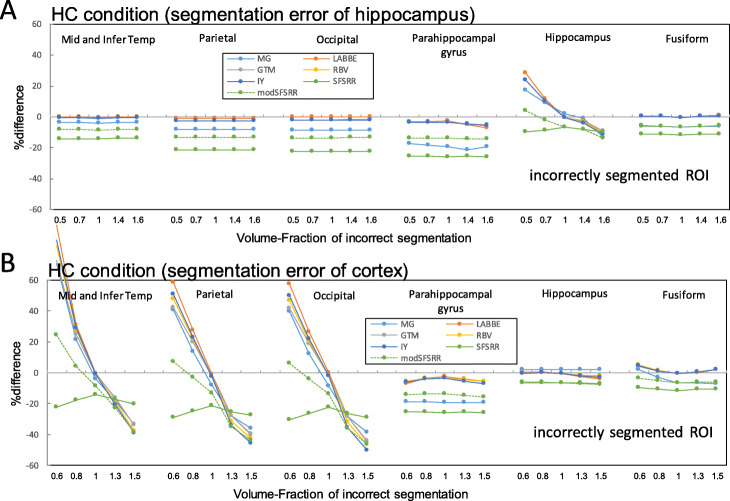
%difference of PVC-SUVRs with segmentation error against true SUVRs. **a** Local (hippocampus) and b global segmentation errors. ROI template was from incorrectly segmented anatomical images

Figure 8 indicates %difference of PVC-SUVRs with segmentation errors of hippocampus and of the global cortex against true SUVRs for the HC condition, where ROI templates were from incorrectly segmented anatomical images. Different tendencies between Figs. 7 and 8 were observed for seven PVC algorithms.

## Discussion

In this study, the properties of error propagation during PVC processing were evaluated. We clearly demonstrated that partial volume correction by any of the developed algorithms improved the quantification of SUVRs for brain THK-imaging under the ideal conditions; on the other hand, error propagation was also observed for all investigated algorithms.

### The necessity of PVC for pathological PET imaging

Under the ideal conditions of PVC, severe partial volume effects (> 30 %difference against the true) were observed in thin or small regions especially in the AD conditions (Fig. 3b). If these regions will be the interest of quantitative analysis or longitudinal analysis, partial volume correction is necessary. Lopez-Gonzalez et al. reported that PV-uncorrected SUVR with the reference region of cerebellar grey matter includes spill-in effect of cerebellar white matter and therefore that PVC would also be in reference region [9]. However, when implementing PVC processing for real clinical data sets, one has to be careful to select an appropriate algorithm of PVC, which has robustness against possible error sources in individual study design.

### Error sources and propagation of PVC algorithms

Errors and their properties of propagation during PVC processing are inevitable in clinical studies. For FWHM mismatch, it is difficult to evaluate accurate shift-variant spatial resolution by using phantom experiments and clinical image reconstruction parameters. Small-bore PET scanners (e.g., small animal or brain-dedicated PET scanner) cause a 1-mm difference in the FWHM between at the center and off-center positions [35]. So, if we assume possible but maximum FWHM mismatch as 1 mm, large errors (> 10%) were still observed in hippocampus and parahippocampal regions (Fig. 4) for all PVC algorithms. In the case of separate PET and MR imaging systems, we have to register MR image to PET image or PET image to MR image for PVC processing. During the registration process, a registration error of one pixel could easily happen due to different distribution and intensities between PET and MR images. In the present study, we found that according to the direction of registration error, the degree of error propagation differed among the regions. As shown in Fig. 5, in the case of registration error in *x*-direction, the parahippocampal gyrus was the region with the largest error propagation, but with registration error in *z*-direction, the fusiform gyrus was the region with the largest error propagation. One needs to be aware of which direction of registration error is most likely to occur. Furthermore, the inaccurately registered ROI template used for the data analysis (Fig. 6) showed larger error propagation properties than true ROI template (Fig. 5). If registration error likely to occur, the use of a ROI template based on inaccurately registered MRI must be avoided in the data analysis.

With segmentation and parcellation of anatomical structure, the inaccurate assignment of anatomical region as segmentation error is also possible error sources on PVC processing [36]. Especially, the hippocampus region for AD patients is the one of the most difficult regions to segment correctly [25]. Furthermore, the cortex is also difficult to segment correctly, especially in elderly subjects, due to cortical atrophy. In this study, we evaluated both regional and global cortex segmentation errors only for the HC conditions. A volume fraction of 0.6 caused overestimation within the small segmented regions because of severe PVE (Fig. 8b) and underestimated in the surrounding regions, and this regional error was averaged by using true ROI template (Fig. 7b). Segmentation error at both hippocampus and global cortex resulted in local error propagations (Fig. 7), where this observation was consistent with results of ^18^F-L-dopa simulation by Frouin et al. [37], and this suggested that it is difficult for us to notice these error propagations.

### Understanding of error propagation in 7 PVCs

Most of the PVC algorithms showed similar error propagation properties, with a few exceptions noted. MG is a voxel-based method for grey matter regions, and the accuracy of the boundary delineation between white and grey matters is important for the PVE correction. MG was sensitive to registration and segmentation errors. Because the contribution of spillover from source region into target region is used for GTM, GTM was also sensitive to registration and segmentation errors. The contribution of spillover from source pixel into target region is also used for LABBE and therefore the error propagation of LABBE was similar to that of GTM. RBV and IY differ in that they use an iterative process; however, similar error propagations were observed between two algorithms.

In SFS-RR and mod, SFS-RR showed different error propagation properties compared to the other algorithms (Figs. 5 and 7). SFS-RR is an image-based algorithm, which weighs the functional versus structural information in the wavelet space based on the dual-tree complex wavelet transform. Thirty-two to 36 % of the structural information was used in the anatomical prior during the resolution recovery [24]. This may have resulted in a lower sensitivity to registration and segmentation errors than the other algorithms. However, under the ideal conditions, SFS-RR showed the worst recovery among the methods (Fig. 3). There were several reported evaluations of the same SFS-RR algorithm using simulation data sets (Monte Carlo or analytical simulation) [24, 38–40]. In particular, similar modest recovery of SFS-RR was also observed in FDG-PET image [24, 38] and striatum regions of [^11^C] Raclopride [40]. Mod SFS-RR improved quantification of the SUVR compared with SFS-RR, but increased the degree of error propagations. Further improvement of the SFS-RR algorithm [41–43] may have a potential for robust clinical use.

### Practical choice of PVC algorithms

In clinical PET study, it would be preferable to use a robust PVC algorithm which suppresses error propagation. In addition to the robustness, the practical choice of PVC algorithms is also related to type of PET scanner and the study design.

For a PET-MRI combined scanner, the registration error should be in theory relatively small, and so the priority would be to use an algorithm that is robust against the segmentation error (e.g., mod. SFS-RR) would be desirable. However, as shown in Fig. 7, segmentation error definitely influences the outcome of PVC for all of the algorithms. Therefore, to avoid error propagation of segmentation error, the accurate and careful segmentation and parcellation of anatomical structure would be important.

For cross-sectional or longitudinal PET study of disease, if progression of disease causes brain atrophy, the degree of error propagation may differ among levels of the progression. Furthermore, pipeline PVC processing and analysis of large data sets, e.g., multicenter study, includes variety of additional error sources, e.g., inter-PET scanner differences and sometimes quality of MRI. In the present study, there were severe error propagations against registration error in the AD condition rather than the HC condition, and segmentation error which is very likely in the case of AD is sensitive for the propagation. So when focusing the investigation of AD progression through PVE-corrected PET images by pipeline analysis, one has to be very careful to avoid error propagation during the PVC processing as much as possible.

In summary, consistent accuracy of the PVC during the progression of disease and robustness against segmentation and any other error sources should motivate both the development of PVC algorithms and the selection of the algorithm for clinical applications.

## Limitation

In this study, we simulated only one HC condition and one AD condition of [^18^F]THK-5351 under the FBP reconstruction algorithm, and the outcome measure was SUVR. So, the %differences, which we reported in this study, may differ with other anatomies and the SUVR distribution/value itself. However, we believe that the present error propagation study of [^18^F]THK-5351 will contribute to both the understanding and prevention of the error propagation phenomena in future clinical PET data analysis. Another limitation is that we only evaluated the unique error sources and did not evaluate multiple error sources in combination. Possible combination may be segmentation error and registration error. Our results suggested that if both regional segmentation error and systematic registration error are simultaneously present in input data sets for PVC processing, most regions except for the few that have been incorrectly segmented will be affected only by registration error. Furthermore, we did not investigate the effect of intrinsic errors in the PET image itself, such as due to inaccurate attenuation correction. If the PET image includes systematic or random errors, these will be propagated to the outcome of the PVC.

While we tried to evaluate many algorithms as reasonably possible, it was not feasible to test all published PVC algorithms. The study of error propagation must continue together with algorithm development. Furthermore, deep learning and machine learning PVC methods are emerging [44], and it remains to be seen, if these approaches automatically suppress error propagation, as this will have great impact on clinical translation.

## Conclusion

We demonstrated error propagation by numerical simulation of THK-PET imaging. The error propagations of seven PVCs algorithms for brain PET imaging with [^18^F]THK-5351 were significant. The robust algorithms for clinical applications must be carefully selected according to the study design of clinical PET data.

## Data Availability

The data that support the findings of this study are available from the corresponding author, MS, upon reasonable request.

## Abbreviations

AD: Alzheimer’s disease
FWHM: Full width at half maximum
GTM: Geometric transfer matrix
HC: Healthy control
IY: Iterative Yang
MG: Müller-Gärtner
MRI: Magnetic resonance imaging
PET: Positron emission tomography
PV: Partial volume
PVC: Partial volume correction
RBV: Regional voxel-based
ROI: Region of interest
SFS-RR: Structural functional synergy for resolution recovery
STIR: Software for Tomographic Image Reconstruction
SUVR: Standardized uptake ratio
